# Fixation options for reconstruction of the greater trochanter in unstable intertrochanteric fracture with arthroplasty

**DOI:** 10.1097/MD.0000000000026395

**Published:** 2021-07-02

**Authors:** Guanning Huang, Mingran Zhang, Zhiguo Qu, Youjia Zhang, Xukai Wang, Wenbo Kang, Minglei Zhang

**Affiliations:** a Department of Orthopedics; b Department of Orthopedic Surgery, Siping Hospital of China Medical University, Siping; c Department of Nuclear Medicine, China-Japan Union Hospital of Jilin University, Changchun, Jilin, P.R. China.

**Keywords:** arthroplasty, intertrochanteric fracture, locking plate, the greater trochanter

## Abstract

**Introduction:**

With the aggravation of population aging, the incidence of intertrochanteric fracture also increases dramatically. Patients are often elderly accompany with severe osteoporosis and various complications. Therefore, we should select an individualized treatment based on the each patient's state. Arthroplasty is recommended for unstable fractures with obvious osteoporosis, ipsilateral femoral head necrosis or arthritis. Rigid fixation of the greater trochanter with arthroplasty is challenging because of the powerful pulling forces created by multiple muscles being transmitted to the greater trochanter. Currently, there are few contemporary literatures on the evaluation of unstable intertrochanteric fracture with efficient fixation of the greater trochanter. Moreover, there is no consensus to choose which implant to immobilize the greater trochanter. The purpose of this study was to review previous literatures and provide a valuable guidance.

**Conclusions:**

The locking plate, which not only provides rigid fixation but also results in lower rate of postoperative complications. However, further prospective randomized and cohort studies are needed.

## 1 Introduction

**Figure 1:**
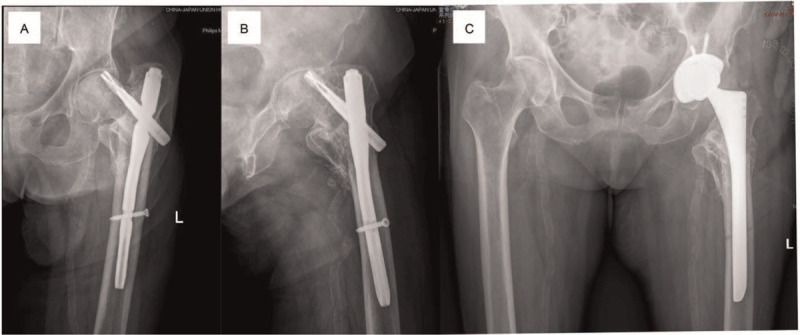
(A, B) AP view and later view showing femoral head lag screw cut-out post surgery. (C) Subsequently, the patient underwent removal of internal fixation and arthroplasty.

Aging population has become a big problem to the worldwide countries, intertrochanteric fractures are the most common type of lower limb fractures in the elderly. Its treatment present a severe challenge to the majority of orthopedists.^[1]^ In China, the intertrochanteric fracture is called the last fracture of life. The incidence of the crowd is often advanced age, multiple medical comorbidities, and accompanied by serious osteoporosis of the elderly. The mortality of no-operative treatment reached up to 34.6%. Osteosynthesis and arthroplasty have been controversial in the treatment of such fractures. However, in recent years, intramedullary fixation has become the gold standard for the treatment of intertrochanteric fractures due to its unique biomechanical advantages. Many literature reports present their views on the choice of implants. Intramedullary fixation (Gamma nail and Intertan double nail system) and Extramedullary fixation (Dynamic hip screw) need shorter operative time, less intraoperative blood loss, and fewer units of blood transfused compare with the arthroplasty.^[2]^ However, these studies often fail to distinguish between stable intertrochanteric fractures and unstable intertrochanteric fractures (AO/OTA type 31-A A2.2, 31-A2.3, and 31-A3.3 and Evans type III IV V). Intramedullary nail and DHS can indeed fulfill rigid fixation for stable intertrochanteric fractures and obtain a good prognosis. When patients accompany with unstable fracture and serious osteoporosis, the higher failure rate of intramedullary fixation including cut out the lag nail (Fig. 1), nonunion, varus displacement, and a series of postoperative complications caused by unable rapid mobilization should not be ignored.^[3]^ Therefore, arthroplasty has been advised as an alternative to internal fixation by some scholars for unstable intertrochanteric fracture.^[4–7]^ The review mainly collects the present literatures in order to explore the indications of arthroplasty for intertrochanteric fracture and the appropriate fixation methods of the greater trochanter.

## 2 Indications of arthroplasty

**Figure 2:**
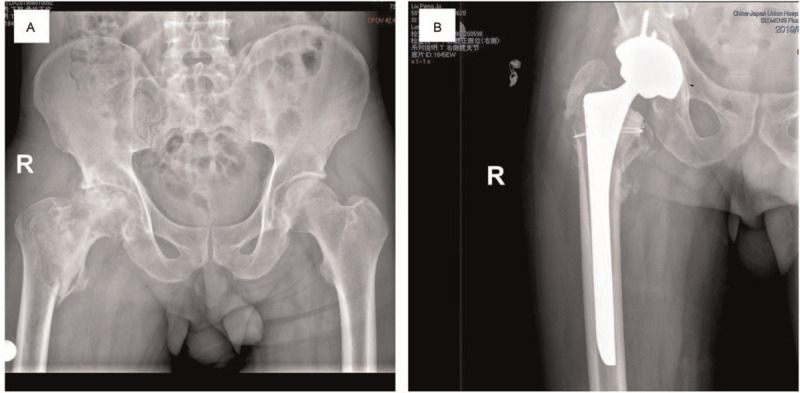
(A) Of an intertrochanteric fracture in a 58-year-old man with preexisting ipsilateral femoral head necrosis. (B) Postoperative radiograph shows he received a THA and immobilized the greater trochanter with metal cable. THA = total hip arthroplasty.

Arthroplasty is suitable for unstable intertrochanteric fracture with the loss of posteromedial cortex support; a fracture pattern that is unlikely to be reduced satisfactorily using a intramedullary nail; with serious osteoporosis; with ipsilateral femoral head necrosis (Fig. 2) or osteoarthritis.^[8]^ Although with longer operative time and more intraoperative blood loss, it lower failure rate of implant and the probability of reoperation, as well as reduced the postoperative complication that can fulfill earlier weight-bearing, providing us with a good choice.^[6]^ One of the technical difficulties in the treatment of the intertrochanteric fracture with arthroplasty is the reconstruction of the greater trochanter. Owing to comminuted fragments and the greater trochanter pulled forward by medial gluteal tendon, the anatomic landmarks are disturbed, so the prudent preoperative plan becomes very important. Another technical problem is lacking proximal metaphyseal support and even lacking diaphyseal support because of osteoporosis, as a result of instability of prosthetic stem. Therefore, cemented prosthesis is usually used for peritrochanteric fractures for better stability, several researches have reported a lower rate of subsidence and periprosthetic fracture with cemented stem.^[9,10]^ On the contrary, Kim et al^[11]^ proposed to use uncemented stem in intertrochanteric fractures, and obtained satisfactory clinical outcomes. In robust and active individuals, those who are at least guaranteed community activity or others with obvious osteoarthritis, many studies have suggested surgery to choose total hip arthroplasty (THA). Because patients who undergo THA show lesser pain, fewer rate of reoperation, and have higher functional score,^[12,13]^ as well as higher risk of dislocation.^[14]^ When face a fragile patient with many medical comorbidities or low-demand older patients, consideration should be given to hemiarthroplasty in order to reduce operative time and blood loss. It should be noted that cemented prosthesis with more blood loss, longer operative time, and the cardiopulmonary complications caused by reaction of bone cement.

## 3 The importance of reconstructing the greater trochanter

When surgeons decide to choose arthroplasty to treat intertrochanteric fractures, rigid fixation of the greater trochanter should be emphasized. The greater trochanter is attachment for the abductor proximally, the vastus lateralis distally, and the short external rotators posteriorly.^[15]^ Those forces act on the greater trochanteric respectively from vertical and anteroposterior plane. In addition to, the abductor will also create a rotational force on the detached trochanter. During normal walking, the greater trochanter carries at least twice the body weight. When climbing stairs, up to 4 times the body weight.^[16]^ If the surgical techniques fail to provide sufficient fixation for the greater trochanter, a serious of postoperative complications will occur. Including fracture fragment displacement; bone malunion; lateral hip pain; hardware failure; reoperations; bursitis; associated limp; abductor weakness, and increase in dislocation rate.^[17]^

## 4 The choice of the internal fixation

### 4.1 Stainless steel wire or wire tension band

**Figure 3:**
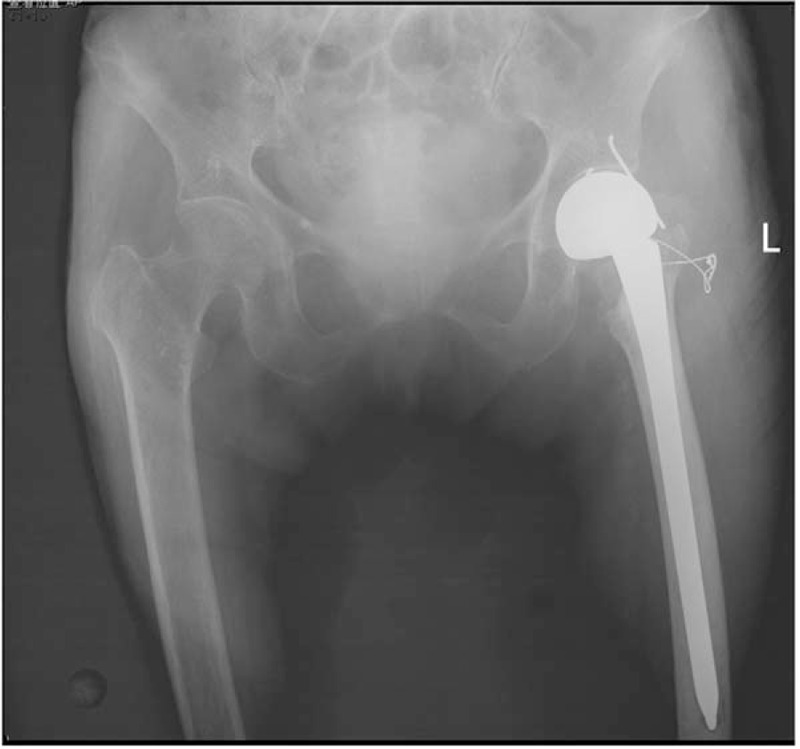
An 89-year-old woman with intertrochanteric fracture, she received a HA and reconstructed the greater trochanter with wire tension band. After 1 week, the kirschner wire pulled out and entered in the acetabulum. HA = hemiarthroplasty.

As the most popular method to fix the greater trochanter, wiring technique were commonly employed in 1960s and 1970s. The relatively simple procedure and mature technology are more acceptable in clinic, whether in the case of a greater trochanteric osteotomy or an intertrochanteric fracture with a hip joint replacement. The advantages of wiring technique include low cost, mature technology, convenient operation, and easy to obtain. However, it also leads to many complications. Frankel et al^[17]^ reported nonunion and fragment displacement rates following wire fixation from 0% to 28%, particularly in revision THA and prior trochanteric nonunion. In vitro studies, Shaw and Daubert^[18]^ have demonstrated that wiring technology with worse resistance to fatigue and lower breaking strength than other fixation methods. So it could not effective to against the pulling strength of the abductor, the biomechanical instability also often leads to the loosening of knots and unequal tension in the loops.^[19–22]^ According to related reports, common complications include loosening and breakage, which may lead to catastrophic consequence (Fig. 3). The migration of broken wire fragment to some locations such as acetabular articulating surface of THA components,^[23]^ the popliteal fossa,^[24]^ and the left side of the heart.^[25]^ Therefore, steel wires seem to more suitable for uncomplicated intertrochanteric fractures. The relatively simple procedure can effectively save the operation time and reduce the economic burden of patients in developing countries. However, wire fixation may not be appropriate for difficult trochanteric fixation, such as revision THA and prior trochanteric nonunion, the choice of the implant needs meticulous consideration before making a decision.

### 4.2 Greater trochanter reattachment (GTR) device

The dall miles cable grip system was introduced in clinical practice in 1980s, this technique was originally used for reattachment of the osteotomized greater trochanter.^[16]^ After decades of continuous improvement, it has developed from the initial first generation to current third generation product. In reviewing the clinic results of monofilament wiring technique found that has many deficiencies, Clarke et al^[21]^ reported the incidences of loss trochanteric position varying from 2.7% to 19.4% and implants breakage from 17.2% to 32%. Under this background, we need a more reliable and effective technique, so the first generation of GTR device was invented. The system consisted of an H-shaped device and 2 horizontal cables. The special hook structure allows it to better to adhere to the surface of the greater trochanter. Two transverse cables drilled through the proximal femur and passed through the bridge of grip. After repeated tightening and compression, the device provided enough fixation and compression of the greater trochanter. In vitro studies, Hersh et al^[26]^ demonstrated that the GTR device had better outcomes and biomechanical properties than other conventional fixation methods. In vivo studies, comparing to other groups the failure rate of internal fixation was lower, the probability of bone union on imaging and postoperative functional results were more satisfactory. However, in a series of subsequent reports, it was suggested that despite GTR device had absolute biomechanical advantages compare with wire fixation, cable breakage, and nonunion rates of 9% to 40%.^[27–29]^ A new claw plate was designed with attention to avoid many complications described in past literatures. The original H-shaped device became an anatomical claw plate. The plate provides fixation below the lesser trochanter. A proximal claw structure allows for grip on the tip of the greater trochanter, transverse, and oblique oriented cables as well as distal teeth sufficiently resisted migration of the plate. Furthermore, anatomic contour improves plate–bone contact. The introduction of second generation GTR system can effectively reconstruct the greater trochanter, increased the rate of the bone union and restore abductor function.^[30,31]^ The first and second generation GTR device still had problem of metal debris from cable wear, which even remained in some frail places. Hop et al^[32]^ discovered metal debris can accelerate wear on the polyethylene surface and loosening of the prosthesis. The emergence of the third generation cable plate system effectively solved these issues. The system included advantages of the early generation systems in addition to improved design that allow for more anatomical construction and improved consistency in tightening and retightening through a uniform cable compression. At this moment, cross-section of the cable tightening demonstrating uniform compression without deformation.^[33]^ However, early generation cable could not be loosened and retightened during the operation. By the time the last cable was tightened, the first cable may have become loose. The only solution to this problem was to cut the residual loose cable and reconstruct another one. But in doing so, we could not guarantee the position or fixation could be stable. The third generation cable plate system introduced a new sleeve that could be crimped to maintain the tension and position of cables so that this system seems to become more reliable because it is specifically designed to provide more uniform tension.

### 4.3 Locking plate

**Figure 4:**
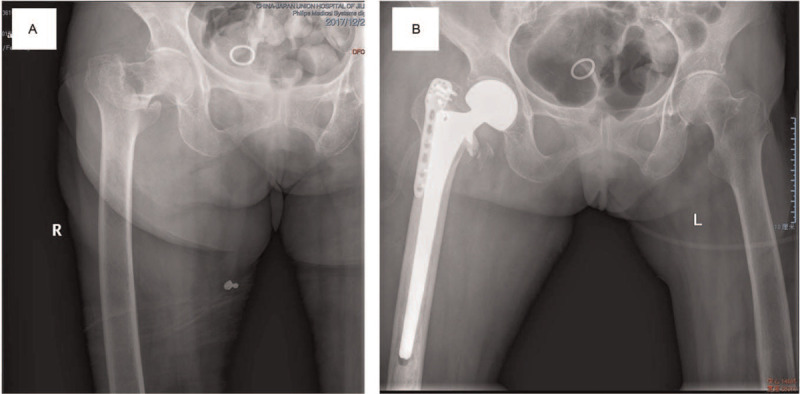
An 84-year-old woman had right hip pain after fall down injury. (A) Pelvis anteroposterior radiograph shows intertrochanteric fracture with obvious osteoporosis. (B) Postoperative radiographic shows she received a HA and the greater trochanter fixed with proximal humeral locking plate. HA = hemiarthroplasty.

As we know, in the late 1990s, locking plate technique was widely used to fracture fixation elsewhere in the body. The locking screw can be locked into the plate. And the locking plate has more stable biomechanical characteristic than traditional plate. Even unicortical bone crews can provide rigid fixation, which is undoubtedly suitable for fixation of greater trochanter (Fig. 4). Multiple literatures has indicated that fixation with locking plate has higher rate of bone union, lower failure rate of internal implants and better postoperative functional scores compared with previous wire and cable-plate system. The main reason of the satisfactory outcome is locking plate can provides better resistance to shear stress and rotational force created by gluteus muscles.^[34,35]^ This theory has also been verified in vitro study.^[36]^

### 4.4 Multifilament cable and nonabsorable suture

Multifilament cable is similar to monofilament wires was widely used to immobilize the greater trochanter past decades. But compared with wires, it can provide a more stable fixation, better resistant to shear and rotational forces made by abductor, reduce the probability of bone nonunion and fracture displacement. However, the complications caused by cables should not be negligible. Concerns have been raised including metal debris from fraying cables and fatigue breakage. Metal debris increased the risk of acetabular loosening and accelerated polyethylene wear.^[32,37]^ Furthermore, multifilament cables cannot be tensioned and compressed by tying into a knot like wires. The knot of titanium cable is easy to unfasten, resulting in failure of internal fixation. As we know, titanium cables are tensioned through cable sleeves before being secured with a crimp.^[38]^ Owing to steel wires and titanium cables had some adverse complications such as fatigue breakage, metal debris generation, and fracture fragment displacement. That has prompted surgeons to use a novel non-absorbable polyester suture of fixation to reduce complications. Especially in recent years, the new ultra-high molecular weight polyethylene fiber cable was introduced. Oe et al^[39]^ had validated that non-absorbable had good clinical results in a multi-institutional research. It not only had lower probability of biological reactivity with soft tissue and less likely to cause deep infection and bursitis, but also had superior tensile strength, fatigue strength, abrasion resistance than other implants.

## 5 Radiographic and functional evaluation

With regard to estimate postoperative the healing of greater trochanter is mainly based on radiographic and functional evaluation. Radiographic evaluation relies on standard anteroposterior pelvis and lateral radiographs respectively at postoperative 2 weeks, 1 month, 3 months, 6 months, 12 months, and annually thereafter. Union could be considered existing if there was osseous continuity between the femur and the greater trochanter and there has not happened displacement of trochanteric fragment or failure of internal implants. Similarly, fiber union could be considered existing if there was radiographic manifestation of nonunion without displacement of the greater trochanter, symptoms of pain and limb. Nonunion could be defined by migration of the greater trochanter, absence osseous continuity between the femur and the greater trochanter, or internal implants failure. Including wire breakage, the kirschner wires of wire tension band pull out, cable abrasion or breakage, screw or plate fracture. Hip functional follow-up used Harris score and koval categories for activity level.^[40]^ Harris score was defined as follow: excellent (90–100 points), good (80–89 points), fair (70–79 points), poor (<70 points). Activity levels were defined as: level I, independent community ambulator, level II, community ambulatory with cane, level III, community ambulator with walker or crutches, level IV, independent household ambulator, level V, household ambulator with cane, level VI, household ambulator with walker or crutches, level VII, nonfunctional ambulatory. Finally, the restoration of hip abductor function deemed to be patient could abduct hip joint against gravity in the lateral decubitus position.

## 6 Summary

**Figure 5:**
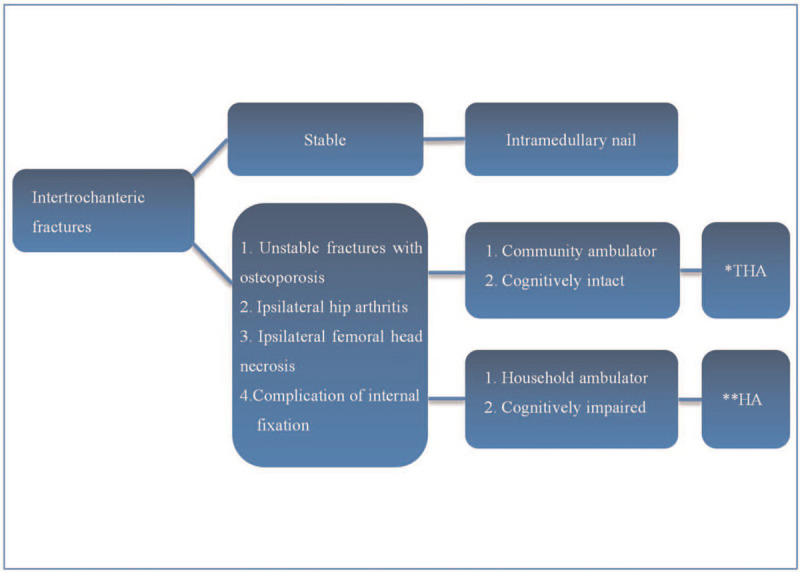
Intertrochanteric fracture treatment flowchart. ∗THA = total hip arthroplasty, ^∗∗^HA = hemiarthroplasty.

Currently, to our knowledge no studies have specifically assessed which fixation method to choose for reconstructing the greater trochanter during arthroplasty for unstable intertrochanteric fracture. With the prevalence of aging population worldwide, the incidence of intertrochanteric fracture in elderly parents is also steeply increasing.^[41]^ In the face of such a fracture, the option of treatment should vary with each individual (Fig. 5). If meet a stable intertrochanteric fracture without significant osteoporosis, intramedullary fixation not only has a biomechanical advantage but also with lesser damage, fewer intraoperative blood loss, and transfusion unit as well as higher long-term Harris functional score.^[5,42]^ However, when treat an unstable pattern fracture with poor bone quality; ipsilateral hip arthritis; ipsilateral avascular necrosis of the femoral head. Hip joint replacement may be a better choice. Although increasing operation time, intraoperative blood loss, blood transfusion unit. It effectively reduces the probability of internal fixation failure and reoperation. In addition, postoperative immediate weight-bearing is important for the recovery of cardiopulmonary function.^[6,43]^ The rigid fixation of greater trochanter has been supported by more and more surgeons, but the choice of fixation is still controversial. Traditional steel wires and titanium is widely used because of its relatively simple procedure and low cost. But the high failure rate of internal fixation also lead orthopedists to seek for other more effective fixation methods. GTR device initially was used as the fixation of the greater trochanter after the greater trochanter osteotomy. After decades continuous improvement, it has been developed into the third generation product and used for arthroplasty in the treatment of unstable intertrochanteric fractures with good clinical results.^[44]^ Lots of literatures have demonstrated that whatever in vivo or in vitro studies, GTR device was more rigid, better resistant to force created by abductor, lower rate of nonunion and fragment displacement than other control groups.^[26,30,31]^ However, there have been also reports of cable wear and metal debris in the GTR device, which can irritate the soft tissue and even fall into acetabular polythene surface,^[32]^ and these complications have not been completely resolved. Oe et al^[39]^ proposed to use high molecular polyethylene non-absorbable cable to immobilize the greater trochanter. Compared with metal cable, it has better fatigue strength, abrasion resistance, and compliance. The emergence of non-absorbable cable effectively solves the complications of titanium cable. In recent years, the appearance of locking plates has provided better stability for fracture fixation. These plates are widely used in various places of the human body, so some scholars began to use locking plates to fix the greater trochanter. Compared with the conventional compressing plates, locking plates with unicortical screws can provide enough stability for fracture site. Furthermore, the complications of this technique have not been demonstrated in the current literatures. The more rigid fixation and lower postoperative complications fully validated the feasibility of locking plates to immobilize the greater trochanter in unstable intertrochanteric fractures, satisfactory results were obtained for all patients in the trial group.^[34–36]^ Actually, many devices can provide stable fixation for the greater trochanter. Surgeons should adequately understand strengths and weaknesses of each implant, because it is crucial to efficient treatment of the fracture. We preferred the locking plate, which not only provides strongest fixation but also results in lower rate of postoperative complications. But further prospective randomized and cohort studies are needed to validate their safety and efficacy.

## Author contributions

**Conceptualization:** Guanning Huang, Youjia Zhang, Minglei Zhang.

**Data curation:** Guanning Huang, Mingran Zhang, Zhiguo Qu, Youjia Zhang, Xukai Wang, Minglei Zhang.

**Formal analysis:** Guanning Huang, Xukai Wang.

**Funding acquisition:** Guanning Huang, Xukai Wang, Minglei Zhang.

**Investigation:** Guanning Huang, Zhiguo Qu, Wenbo Kang.

**Methodology:** Mingran Zhang, Zhiguo Qu.

**Project administration:** Youjia Zhang.

**Resources:** Guanning Huang, Xukai Wang, Wenbo Kang, Minglei Zhang.

**Software:** Mingran Zhang, Zhiguo Qu, Youjia Zhang, Wenbo Kang.

**Supervision:** Guanning Huang, Mingran Zhang.

**Validation:** Mingran Zhang, Zhiguo Qu, Youjia Zhang.

**Visualization:** Guanning Huang, Mingran Zhang, Zhiguo Qu.

**Writing – original draft:** Guanning Huang.

**Writing – review & editing:** Guanning Huang, Mingran Zhang, Minglei Zhang.
